# Safer sex practices among newly diagnosed HIV-positive men who have sex with men in China: results from an ethnographic study

**DOI:** 10.1080/17482631.2017.1335167

**Published:** 2017-06-15

**Authors:** Haochu Li, Andrea Sankar, Eleanor Holroyd, Baofa Jiang

**Affiliations:** ^a^ School of Public Health, Shandong University, Jinan, China; ^b^ Department of Anthropology, Wayne State University, Detroit, MI, USA; ^c^ School of Clinical Sciences, Auckland University of Technology, Auckland, New Zealand

**Keywords:** Safer sex, new HIV diagnosis, men who have sex with men, China

## Abstract

The study reported here sought to understand the rationales of safer sex practices adopted by newly diagnosed HIV-positive men who have sex with men (MSM). Guided by a socio-ecological framework, an ethnography was conducted among newly diagnosed HIV-positive MSM. In-depth interviews and participant observation were employed to produce an account of the social and cultural settings that was faithful to the perspectives of participants. A total of 31 participants with diverse backgrounds were recruited in a southern city of China. Participant observation was conducted in local healthcare settings, MSM venues, and NGO offices. Most participants (24/31) reported stopping unprotected anal intercourse (UAI) immediately after being diagnosed as HIV-positive. Factors associated with safer sex practices were identified at both individual and environmental levels, including self-protection, establishment of self-esteem, dignity, altruism and reciprocity, disease experience as a source of personal growth, and organizational culture and values. Newly diagnosed HIV-positive MSM navigate their sexual practices within the context of multiple competing factors. Implications for sustained behaviour change enabling safer sex practices include stimulating survival instinct, facilitating safer sex decision making, motivating and facilitating personal growth, and encouraging volunteerism to promote intentional activities for safer sex practices.

## Introduction

To date, significant progress has been achieved in identifying the psychological correlations associated with safer sex practices. Some studies identify such characteristics as cognition, intention, emotion, attitude, skills, coping, self-efficacy and motivation associated with safer sex practices of people living with HIV (PWLH) (Inoue et al., 2006; Kalichman, Williams, Cherry, Belcher, & Nachimson, 1998; Rotheram-Borus, Reid, Rosario, & Kasen, 1995; Schutz et al., 2011). Researchers found that group level safer sex interventions that have successfully targeted self-efficacy and motivation have improved PLWH’s safer sex practices (Kalichman et al., 2001; Wingood et al., 2004). HIV positive men who disclosed their seropositive status and explicitly discussed safer sex with their at-risk partners had a significantly higher prevalence of protected anal or vaginal intercourse than the ones who disclosed their seropositivity only (Crepaz & Marks, 2003).

A second area of progress has been in the domain of identifying characteristics of sexual encounters associated with safer sex practices. Factors such as the number of sex partners, concurrency, duration, partner type, and serostatus concordance are associated with the practice of safer sex (Robinson, Bockting, Rosser, Miner, & Coleman, 2002). Partner type and characteristics can also influence whether condom use behaviour is consistent with the norms of personal responsibility in safer sex practices (Van Kesteren, Hospers, Kok, & Van Empelen, 2005) and condom use with multiple and/or anonymous partners (Valdiserri et al., 1988). Furthermore, there is a lack of association between restricting oneself to a single sexual partner and condom use (Reiss & Leik, 1989). Women with high levels of relationship power (i.e., relationship control and decision making dominance) were five times more likely to use condoms, compared to women with low levels of relationship power (Pulerwitz, Amaro, De Jong, Gortmarker, & Rudd, 2002).

A third area concerns the socio-environmental factors, such as peer norms (i.e., descriptive norms, injunctive norms, and peer pressure), social support, stigma, and socioeconomic factors (Kalichman et al., 1998). Some studies suggest that norms mediate the effects of other psychosocial factors (e.g., attitudinal beliefs, self-efficacy, awareness consequences, and ascription responsibility) on condom use for anal sex (Van Kesteren, Hospers, Van Empelen, Van Breukelen, & Kok, 2007).

Sexual behaviour is inherently social in nature and is often determined by constituents of networks. Social network components may activate or strengthen condom use norms within networks, which in turn, determine consistent condom use among men who have sex with men (MSM) (Liu et al., 2009). However, the nature of the subjective experience has received little attention, limiting contextual understanding of safer sex practices, which means avoiding condomless sex or using masturbation as an alternative. In a motivational interview-based safer sex intervention in the USA, a majority of clients chose to discuss issues of communication, relationships, and self-care, suggesting that safer sex practices are experienced within people’s social lives (Golin et al., 2007). Attention to these factors is supported by the sexual health model which posits that one’s behaviours, values and emotions are integrated with a person’s wider personality structure and self-definition (Robinson et al., 2002), suggesting that it is necessary to situate sexual practices in both individual and environmental contexts (Neville & Adams, 2016).

To better understand the full range of factors associated with safer sex practices, the current study adopts a qualitative approach guided by the socio-ecological model (McLeroy, Bibeau, Steckler, & Glanz, 1988) to examine both individual and social environmental factors influencing safer sex practices. To date, studies of safer sex practices are still limited in China. The subjective experiences of safer sex practices among newly diagnosed HIV-positive MSM have been largely ignored. Understanding the socio-ecological context of safer sex practices from the perspective of newly diagnosed HIV-positive MSM will provide an emic insight into the positive aspects of sex practices in order to improve secondary HIV prevention programmes.

## Methods

### Settings and research design

In 2010, Shenzhen had a population of 13.1 million and over 80% of the population were internal migrants who had migrated from other parts of China with or without household registration in Shenzhen (Shenzhen City Government General Office, 2011). It is estimated that there were approximately 100,000–200,000 MSM living in Shenzhen (Xie et al., 2010). Ethnographic fieldwork was conducted by the first author in Shenzhen from January to September 2010 in collaboration with a local non-government organization (NGO). Ethnography is a qualitative methodology that employs participant observation and in-depth interviews to explore local meanings, practices, values, and beliefs characteristic of a specific cultural group or setting (Blommaert, 2006; Green & Thorogood, 2009). The NGO worked closely with the Shenzhen Centre for Disease Prevention and Control (Shenzhen CDC) to provide HIV/AIDS prevention services to MSM and was well-connected to some grass root groups of the PLWH. In addition a life-profile approach was used to focus on individual life narratives that provided background and context to an individual’s contemporary community life (McCance, McKenna, & Boore, 2001). While risky sex is a significant topic and of major concern in current HIV prevention studies, it’s equally important to understand the process and ways in which newly diagnosed HIV-positive MSM practise safer sex. The current paper, therefore, emphasizes these men’s subjective experience of safer sex practices subsequent to being newly diagnosed as HIV positive.

### Participants

We used purposive sampling to recruit participants with diverse background characteristics (Table I). The respondents recruited covered different types of HIV positive MSM in Shenzhen purposively based on different income levels, occupations, time of becoming infected (ranging from just diagnosed HIV positive to diagnosed in the 6 months), sexual identities, ages, sources of migration, and degrees of involvement in HIV positive groups. Eligibility criteria included: being 18 years of age or older, diagnosed as HIV-positive in the last six months (newly diagnosed) (Sena, Torrone, Leone, Foust, & Hightow-Weidman, 2008), self-identification as a man who has sex with men, and willingness and ability to provide written informed consent. Data about adaptation to the new HIV diagnosis reached saturation when a total of 31 eligible participants completed the repeated in-depth interviews (an initial interview and a follow-up interview) in a three-month interval over the course of fieldwork. All participants were internal migrants. A detailed interview guideline was developed. In the first round of interviews, the guideline focused on more general information, drawing out the contextual account of the life circumstances where newly diagnosed HIV-positive MSM were situated. This included data of background information of the MSM communities, networks, health care settings, and related authorities; their life history; knowledge and attitude about services, and satisfaction of stakeholders (i.e., health care workers, volunteers, and peers). Based on a quick analysis of the data obtained from the first interviews, some specific questions related to sexual behaviour and relationships were probed in depth. This repeated in-depth interview approach provided rich information, which could be used for further explanations or interpretations.

**Table I.** Summary of the characteristics of the newly diagnosed HIV-positive MSM who took part in repeated in-depth interviews.

Characteristics	Repeated Interviews	Total % (*N* = 31)
**Age**		
18–25	8	25.81
26–35	19	61.29
36–40	4	12.90
**Duration since Diagnosis**		
1–2 months	15	48.39
3–4 months	11	35.45
5–6 months	5	16.13
**Occupations**		
Office	10	32.26
Service/seller	5	16.13
Technician	9	29.03
Labourer	4	12.90
Sex worker	1	3.23
Jobless	2	6.45
**Education**		
College	8	25.81
High/technological school	17	54.84
Secondary school	6	19.35
**Monthly Income (CNY^1^)**		
More than 6000	3	9.68
3000–6000	12	38.71
Less than 3000	14	45.16
No income	2	6.45
**Sexual Identity**		
Homosexual	28	90.32
Bisexual	3	9.68

^1^One U.S dollar was equal to 6.80 CNY in 2010.

### Procedures

The collaborating NGO approached prospective participants through their established networks. Written informed consent was obtained, assuring data confidentiality, use of pseudonyms, safe storage of the data, audio-taping, and the right to withdraw at any time without being disadvantaged. Second interviews were conducted for each participant three months after the initial interview. Translated verbatim data from the initial interview was read through first, and emerging themes, missing points from the subsequent interview guide, or incomplete information were explored in the second interview. Each interview lasted between 90 min to 2 h and was conducted in Mandarin in a private room in the NGO’s office. We gave participants a cash reimbursement (RMB 500 totally, approximately USD 80) to compensate for their time spent undertaking the repeated interviews. The first author also conducted participant observations in MSM venues, NGO offices, and local healthcare settings. Twelve men from our main study accepted the first author’s engagement in their daily lives, such as visiting their homes or work places, and hanging out with them. The Survey and Behavioural Research Ethics Committee in The Chinese University of Hong Kong granted ethics approval.

### Data analysis

A general inductive approach was used to analyse the raw data, starting with a transcription of the digital recordings into written form resulting in a list of themes (Thomas, 2006). The tape-recorded interviews were transcribed verbatim into Chinese. We employed thematic content analysis (Bernard, 2011; Green & Thorogood, 2009) concurrently with data collection in order to capture emerging themes. The first step was familiarization and immersion in the raw data. The analysis involved multiple times of reading of transcripts and field notes and identifying recurring themes. The second step was identifying a thematic framework. This was carried out by drawing on *a priori* issues and questions derived from the aims and objectives of the study as well as issues raised by the respondents themselves and views or experiences that recur in the data. The authors discussed areas of emerging importance and agreed on a set of preliminary codes. The third step was applying the thematic framework (coding scheme) systematically to all the data in textual form by annotating the transcripts with labels (names of the codes) from the coding scheme. Guided by the adapted socio-ecological framework, we grouped together informative quotes relevant to the themes, such as self-protection, self-esteem, dignity, altruism and reciprocity, personal growth, and volunteerism. The fourth step was synthesizing the data according to the appropriate part of the thematic framework to which they related. Finally, we refined the thematic structures based on ethnographic observations and collective discussion. The first author translated the transcripts into English, and the co-authors crosschecked the accuracy and completeness of translations.

## Results

Among the 31 participants, most of whom (61%) were aged between 26–35 years, and almost half were diagnosed as HIV positive within 2 months preceding the interviews. Most of the participants (74%) had an education level lower than college completion. Most of them had a monthly income less then RMB 6000 (USD 882). Most participants (24/31) reported stopping UAI immediately after being diagnosed as HIV-positive. Multiple factors associated with safer sex practices among these participants were identified, emphasizing self-protection, establishment of self-esteem, dignity, altruism and reciprocity, disease experience as a source of personal growth, and organizational culture and values.

### Self-protection

To “protect oneself” (a survival instinct) was a generally reported reason for safer sex practices. The infection of HIV was seen as a serious threat to the participants’ health, and they worried about being infected by other viruses or cross-infections. A participant, Zhang, gave the following rationale for practising safer sex:

Using no condom when having sex will cause cross-infection… Because I want to ‘huo xia qu’ (survive). It’s a simple reason.

Self-protection was a key motivator for safer sex practices as participants sought to prevent further deterioration of their health. Those participants who expressed a strong desire to protect themselves generally felt they had good life management skills and an awareness of STDs, anal diseases, cross-infection and complicating diseases when reporting that they practised safer sex consistently. A participant, Chen, said,

I would not deliberately hurt others. I would not do that. It’s also a kind of self-protection. No matter how unlucky I am, it’s my own business and it has no relation to others. I would not take revenge on someone, since it will bring me stress.

“Jiao cha gan ran” (cross-infection) was a term frequently used by CDC staff, medical doctors, volunteers in NGOs and some HIV-positive peers. It was cited as a reason to prompt newly diagnosed HIV-positive MSM to use condoms when they had sex with others. A participant, Hua, testified:

After getting HIV infected, I have been especially afraid of being infected by other viruses since my immunity is very low now….As the doctor said, the viruses we get infected with are different. If no condom is used, it’s easy to have cross-infection, since our immunity is very weak. …Therefore we must use condoms and reduce the risk of getting infected by other viruses.

In this regard, the motivation behind avoiding infection by any virus again and practising self-protection was their instinct to live and to avoid suffering. Furthermore, self-protection was not only about avoiding health deterioration, but also about avoiding “troubles”. Some participants worried other men would take revenge on their sex partners who were regarded as having transmitted HIV to them. Safer sex protected them from getting into this trouble. A participant, Xu, said:

Sometimes I really want to have sex, but I dare not do so. Once I transmit the virus to them, I will really be in trouble. I therefore need to control myself. If I cannot control it, I will then overcome it by myself [masturbation]. I would not do it [have sex] without condoms. Even though I don’t like to use condoms, I dare not have sex with them without condoms…I am afraid and don’t want to hurt others. It’s real.

### Establishing self-esteem, dignity, altruism, and reciprocity

The practice of safer sex was described as building self-esteem (e.g., being full of love) and self-expectation to be a moral person (e.g., being strong, mature, and respectable). A participant, Zhou, said:

Before I made love with others, I did not disclose to others that I am aizibing (AIDS). But I reminded others to use condoms. …If partners insisted on using no condoms, I gave up having sex [with them]. …I am a person full of love; I become stronger, more mature and respectable… I cherish life and realize its importance.

Consistent safer sex practices also included negotiations with negative public social stereotypical thinking of PLWH Participants felt confidence and self-respect after they disciplined themselves not to hurt others through such practices as using condoms or exercising abstinence. A participant named Qi, who believed in Christ, said:

I feel my heart is kind, and my heart will not hurt others…to use my lovely heart to love others, I feel it is really good, turning from evil to good.

Some participants strongly insisted on using condoms and even disclosed their status to their sex partners in order to teach them the importance of consistent condom use. This practice could serve to build up stronger relationships between the participants and their sex partners. Zhao was such a participant and obtained deep appreciation from his sex partner. Zhao said:

Later I chatted with him on QQ (an online platform). I asked ‘why don’t you use condoms?’ and he said he didn’t like it, condoms were not convenient, and it was not his habit. I said you would get AIDS sooner or later. He said it’s impossible since he is not promiscuous. I said the impossible thing is just around you; I am also not promiscuous; I am just around you and I am ‘A’ (got AIDS). At last, I said you must use condoms in the future. … He really appreciates me.

The desire to protect others was experienced as a kind of altruism; however, participants described this not only as about helping others, but also as essentially a kind of self-help. They understood this as creating a kind of reciprocity. A participant, Shen, said:

I feel I don’t want to do that [take revenge on others or society]. I feel that it is meaningless. What benefits will I obtain if I hurt others? None at all. If I go help others, I may obtain a kind of comfort. …I may help myself if I help others, since I can have a happy mood, and I can obtain peace of mind.

### The disease experience as a source of personal growth

Some participants questioned themselves and were reflective about their previous life experiences. They felt they had learned some lessons from becoming HIV positive. These translated into such behaviours as getting to know more about HIV and condom use, building up emotional attachments and enduring relationships, giving up unhealthy lifestyles, and caring more about their families and people around them. They found out something positive in their sufferings caused by HIV, and took it as an opportunity to facilitate personal growth.

Jiang was a typical case. After becoming HIV positive, his boyfriend still accepted him and they then set up a couple relationship and lived together. Jiang said:

If I just want to seek sex, I would not look for a stable boyfriend…I really want to have a stable boyfriend to fulfil my need of emotional attachment, and sex is not the most important thing.

Jiang reduced his anal sex practice with his boyfriend and consistently used condoms, since he worried about transmitting HIV. He also totally changed his lifestyle. He described his previous life as “mi lan” (erosive) and “fu zao” (volatile and impetuous). He was reflective that he had previously thought he had some “zhi ben” (advantages or superiorities), such as being well-educated, having a good income and being handsome, and he would not think about setting up an enduring relationship. But now he felt he had nothing and he wanted to make a difference and have meaning in his life. He said:

If it were not Liu (his boyfriend), others would have left me, or I needed to lie to them to hide [my seropositive status] … selfishly speaking, I have no choice. But this choice is just the one I need.

Another participant, Yang, described his positive changes as the following:

My personality has changed. Previously I felt ‘wu suo wei’ (indifferent) to everything…I was free to do everything as I liked. After I got this disease, the most important change I have undergone is treating my family better…spending much more time with my parents and chatting with them. … My mentality is balanced, not so irritable as in the past… and my life situation has changed as well. After work, I like to go back home earlier, or do physical exercises, or go to the gym, or stay at home with my parents. …I changed many bad habits, caring more about people around me, cherishing everything that I have.

Yang attributed his positive changes to the following reasons:

Maybe the biggest reason is that I am restricted by this disease and I cannot screw around. Secondly, after taking these medicines (ART), there has been some restriction, such as not staying up late or not working too hard. After I have these two basic changes, many things gradually change in this direction automatically.

The above cases show that participants were reflective and had learned from their disease experiences and some were experiencing personal growth.

### Organizational culture and values

Volunteerism has been widely accepted in MSM communities in Shenzhen. Some participants were volunteers (11/31). They had learned about HIV/AIDS from volunteer organizations and developed to take on the collective identity of being a volunteer advocating for negotiating condom use with sex partners. The participant, Zhao, utilized volunteer work as a strategy to negotiate condom use and to avoid disclosure of his seropositive status to casual sex partners. He described:

I spoke to him (sex partner). Because I have a lot of condoms and booklets in my bag, I said ‘I am a volunteer. I pay much attention to HIV/AIDS. I don’t know whether you have diseases and you also don’t know whether I have diseases; therefore it’s better to use condoms’.

The participant, Hua, discussed HIV issues with his friends and pushed them to use condoms. He sometimes faced the question of “why do you know about that (HIV)?” He then explained:

I am a volunteer and I take part in activities in ‘YGL’ (the association of volunteers)…The HIV issue has become serious and we need to use condoms.

Volunteers also promoted the identity of tongzhi (gay) in the MSM community. The collective identity of tongzhi was used to encourage condom use by some participants. A participant, Qin said:

If he wants to use no condoms, I make it definitely clear that we must use condoms… I say it’s for our safety…as tongzhi, we must use condoms when we do it (have sex).

This is not a single case. During my fieldwork, a volunteer named Murong chatted with me in a tongzhi bar. It was my first time to meet him and he therefore treated me as a general client at that moment. He distributed a package of condoms to me and said, “As a tongzhi, it’s our responsibility to use condoms”.

During the first author’s fieldwork, peer pressure/influence showed positive effects on intentional condom use among close friends. Jiang and Liu were a sero-discordant couple in the volunteer circle. They practised condom use consistently. Tu, a volunteer and a close friend of Jiang and Liu, admitted that he had UAI previously, but was influenced by Jiang and Liu, and started practising consistent condom use with his boyfriend as well. Shuai was another volunteer, who did not use condoms with his boyfriend. Later, he was criticized to the point of crying by the leader of the volunteer group and other volunteers also disagreed with this behaviour. In Ping’s apartment, it was observed that Ping and his new boyfriend were discussing condom use, and later, Ping made his boyfriend to use condoms.

Some participants adapted to the diagnosis of HIV by taking action to contribute to the MSM community. They clearly self-identified as being HIV-positive, and they wanted to help others through volunteer work. A participant, Hei, set up a QQ group and chatted with newcomers online, providing them information and psychological support. Another participant, Zhu, took part in SRW (a grassroots NGO) social initiative campaigns against HIV/AIDS and presented himself as an example of PLWH in a seminar in front of many middle school students. The participant, Shen, said:

I rely on myself…comfort myself, and adjust myself…I therefore think that for those newly diagnosed HIV-positive people… some of them are very young, or have just graduated from school… how can they go through their later life? I therefore want to help them…I have experienced a lot indeed and have a better coping capability.

The above cases show that expectations and judgments from significant others (e.g., peers and friends) played an important role in social surveillance. These informed the development of new social norms of valuing protecting others. The activities organized in MSM communities or NGOs encouraged participants to initiate positive health interactions with their peers and friends.

## Discussion

Our study has explored positive experiences of safer sex practices from newly diagnosed HIV-positive MSM. Multiple factors of self-protection, establishment of self-esteem, dignity, altruism and reciprocity, personal growth, and volunteerism have been highlighted as influential in safer sex practices. Through discussion of the aforementioned positive factors, we came up with four strategies to promote safer sex practices among HIV-positive MSM: (1) stimulating survival instinct, (2) facilitating safer sex decision making, (3) motivating and facilitating personal growth, and (4) encouraging volunteerism to promote intentional activities for safer sex practices.

### Stimulating survival instinct

Our findings show that the initiation of self-protection is an important indicator that these men have adjusted to their HIV diagnosis by better managing their lives and by learning about cross-infection. A practical implication is that in post-test counselling, counsellors or healthcare workers should stimulate PLWH’s survival instinct in order to encourage them to practise self-protection. The survival instinct is future-oriented, and includes hope of survival, disease management, and skills acquisition in managing social relationships. Informed by cognitive adaption theory (Taylor, 1983), counsellors or healthcare workers should facilitate HIV-positive MSM to understand the reason they acquired HIV, the impact HIV infection would have on them, the way to manage it, and how to make themselves feel better.

### Moral personhood: facilitating safer sex decision making

Our findings show that many HIV-positive MSM saw themselves as a good person, which serve as motivations of safer sex practices (O’Leary & Wolitski, 2009). They disciplined themselves to behave in a moral fashion, which in turn affirmed a positive self-evaluation. It should be an important strategy to recruit peer activists to share their world views and adjustment process to newly diagnosed HIV-positive MSM in their follow-up counselling or services. One of the emphases should be on promoting values of altruism and reciprocity. Peer activists could share how altruism, such as practising safer sex and helping others, can result in self-help and reciprocated behaviours among HIV-positive MSM and their partners. Another emphasis is that peer activists should use their own experiences on the benefit of helping others, through which HIV-positive MSM would be directed to make a rational decision of practising safer sex.

### Motivating and facilitating personal growth

Our findings show that these men were reflective, critical, learning from their previous life experiences and actively readjusting. As Janoff-Bulman (2004) argued, a person can be regarded as experiencing growth when he learns about himself, moving from concerns about the meaning of life to the creation of meaning (i.e., value and worth) in life. Motivational interviewing, as a way to help people work through ambivalence and commit to positive change (Golin et al., 2007; Hettema, Steele, & Miller, 2005), should be utilized as a practical strategy to facilitate HIV-positive MSM to reach personal growth in follow-up counselling or services. Healthcare professionals and peer activists should be provided with related training and work together to motivate and facilitate HIV-positive MSM to make positive changes in their lives, such as safer sex practices.

### Encouraging volunteerism to promote intentional activities

Volunteerism has endowed volunteers with “xian jin xing” (advanced nature/civilization), which is a powerful discourse in contemporary China. To be a volunteer means that you need to be civilized/advanced and other people will also see you this way. Condom use has been constructed as a civilized/advanced behaviour and a norm of responsibility in MSM volunteer circles. Another strong discourse in volunteerism is “you ai xin” (affectionate). Those active volunteers who are also HIV positive have internalized this discourse that “I am a kind person”. As Burke (2017) pointed out that people act to verify or confirm their identities, these volunteers also developed skills for negotiating condom use with their partners and took actions to contribute to the MSM community. Having social support for safer sex and positive peer norms has been shown to predict avoidance of U.A.I among HIV-positive MSM (Forney, 2008). Volunteering can contribute to MSM’s social capital with the positive result of increasing self-esteem, a sense of empowerment, and safer sex behaviours (Ramirez-Vallues & Brown, 2003). Activating volunteerism to promote intentional activities (e.g., safer sex practices) has widely been used in positive psychology as an important measure of intervention (Sin & Lyubomirsky, 2009).

## Limitations

There are limitations in this study. First, the consented use of a digital recorder may have obstructed some participants from openly talking about their negative views of sexual practices. Second, the relationship between local NGOs or HIV positive groups was sensitive, which constituted an obstacle for the first author in gaining access into the different groups of HIV-positive MSM. In addition, we did not recruit participants who had no contact with healthcare workers or volunteers. These hardest-to-reach HIV-positive MSM may have different views of safer sex. Third, although an interview outline was provided, not all participants received exactly the same series of questions in the same order. Data inconsistency could therefore be a potential problem.

## Conclusion

The current study contributes to our understanding of the newly diagnosed HIV-positive MSM’s practices of safer sex with a Chinese context and explores a dynamic framework examining safer sex practices from the perspective of these men. Interventions based on both personal and environmental levels, such as motivational interviewing in post-test counselling and encouraging volunteerism among PLWH, are crucial for sustained safer sex practices. In order to broaden the understanding of psycho-social determinants of condom use behaviours among MSM, it would be important to reproduce this study in different cultural, racial and ethnic groups.
